# Data from routine meat inspection is a poor indicator of the prevalence of tail lesions in undocked pigs

**DOI:** 10.1186/s40813-020-00149-z

**Published:** 2020-04-14

**Authors:** Hanne Kongsted, Leslie Foldager, Jan Tind Sørensen

**Affiliations:** 1 Department of Animal Science, Aarhus University, Blichers Allé 20, DK-8830 Tjele, Denmark; 2 Bioinformatics Research Centre, Aarhus University, C.F. Møllers Allé 8, DK-8000 Aarhus C, Denmark

**Keywords:** Tail lesions, Undocked pigs, Abattoir registrations, Clinical registrations

## Abstract

We investigated the prevalence of tail lesions in batches of undocked slaughter pigs in herds just before delivery to an abattoir. At the abattoir, dehaired and scalded carcasses were submitted to routine meat inspection which included recording of tail lesions. The purpose of the study was to investigate the relationship between clinically and abattoir- detected tail lesions in undocked pigs.

During visits in 15 label-production herds, 2346 slaughter pigs from 24 batches were examined. Tail lesions were registered as mild healed, mild unhealed or severe. The median prevalence of the three categories in batches was 13, 9 and 6%, respectively. At the abattoir, tails were evaluated by public inspectors. Between 0 and 10% of pigs within batches (median: 1%) were registered with tail lesions at the abattoir. A linear regression model was used to compare the proportions of severe tail lesions registered in each batch within the herds with the proportions registered at the abattoir. We applied a leave-one-batch-out internal cross-validation on the model in order to explore a systematic relationship. The mean absolute difference between the predicted and the observed proportion was 9%-points. The coefficient of determination (r^2^) was 0.006.

Our results indicate that there is no systematic relationship between clinically and abattoir-registered tail lesions in undocked pigs. Thus, abattoir registrations as carried out in the present study did not mirror the clinical situation properly. If meat inspection recordings should be used to reflect tail lesions in the herds, efforts must be undertaken to ensure a positive correlation between the two.

Thus, abattoir registrations used as an indicator of tail bite prevalence in herds are currently not reliable.

Tail lesions resulting from tail biting is a major welfare issue in commercially produced pigs. Routine tail docking is banned in the EU and there is increasing pressure for stricter implementation of this legislation [1]. A reliable abattoir-based system for monitoring the prevalence of tail lesions in herds raising undocked pigs is of interest for both farmers and authorities. In Denmark, public abattoir inspectors record tail lesions as part of a standardized routine meat inspection protocol [2]. Meat inspection protocols were originally designed to detect diseases posing a risk to public health. Nowadays, meat inspection data is widely used for different purposes including assessing animal welfare (for a review, see [3]). However, using abattoir data for monitoring animal health and welfare in herds is not straightforward. Limited data on the performance of routine meat inspection with reference to clinically obtained registrations is available [4–6]. Furthermore, the majority of studies investigated pigs with docked tails.

The aim of this study was to investigate, if current routine meat inspection recording of tail lesions in undocked slaughter pigs is a useful tool for monitoring tail biting problems in label-production herds^1^ housing undocked pigs. We investigated if the prevalence of tail lesions detected at the abattoir reflected the prevalence of severe lesions detected in live pigs in the herds just before slaughter.

During two cold seasons in 2017 and 2018, 24 batches of undocked slaughter pigs from 15 Danish label-production herds were clinically examined for tail lesions just before delivery to the abattoir. Prior to the visits, farmers were instructed either to colour mark pigs intended for delivery within the following week or to separate them in specific pens. During the visits, the same person (HK) examined 2346 pigs (15 to 218 pigs per batch) within their pens. No physical restriction was necessary, as the pigs behaved calmly and allowed the examiner to closely inspect and palpate tails (palpation was done when necessary to distinguish between dirt and crusts).

In the herds, tails were scored in four categories: Normal (intact skin, tip of tail with un-abrupt ending and hair on the entire surface), mild healed lesion (intact skin, tip of tail with sharp and abrupt ending and with no hair on the most distal part), mild unhealed lesion (un intact skin, tip of tail with sharp and abrupt ending, and no hair on the most distal part) and severe lesion (un intact skin and swelling and/or marked loss of tissue). As shown in Fig. 1, the prevalence of tail lesions detected clinically differed markedly between batches. Overall, tails with mild healed lesions were quite prevalent (median: 13% per batch), whereas mild unhealed and severe lesions were seen in fewer animals (median: 9 and 6% per batch, respectively). As seen from the categorisation, acute cases with swelling were a subset within the severe category. The proportion of acute lesions out of the total number of lesions in the batches varied with min: 0%, median: 2% and max: 13%.

**Fig. 1:**
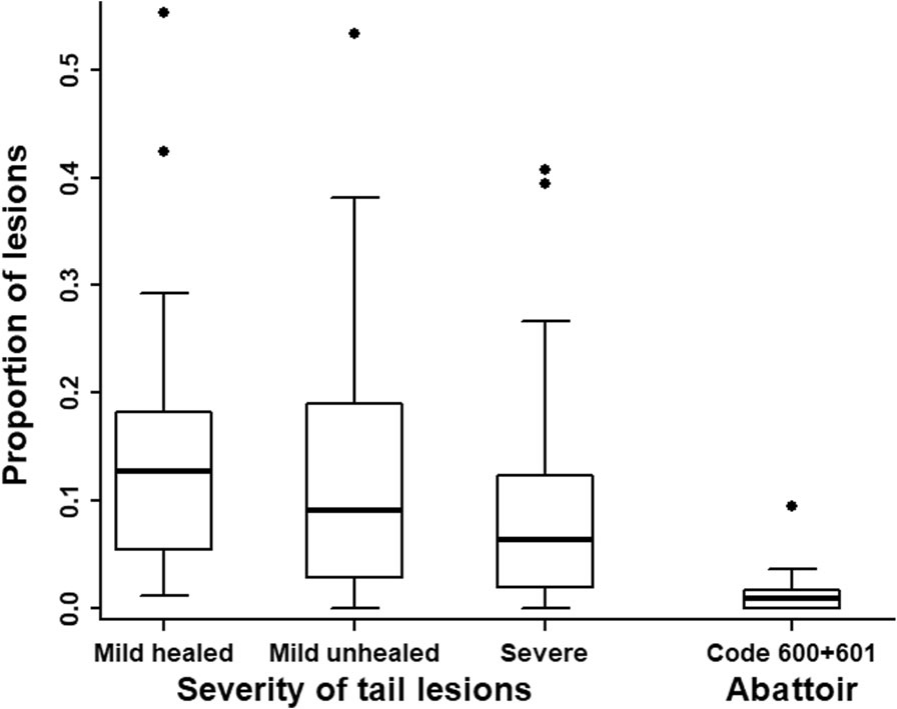
Tail lesions detected at the clinical examination in the herds (left) and at the abattoir (right). Box-and whisker plots of the proportion of pigs with Mild healed, Mild unhealed and Severe lesions in 24 batches of slaughter pigs just before slaughter

All pigs were slaughtered at the same Danish abattoir, and thereby we avoided inter-abattoir variation. No information on the number, identity or training of individual meat inspectors was available. In total, 2449 pigs were inspected at the abattoir – thus, in a few cases, more pigs than marked (and examined clinically) in the herds were sent to the abattoir. At the abattoir, standard meat inspection was carried out by inspectors using government specified codes [2]. Two codes were used for registration of tail lesion: Code 600: “Tail bite, local, limited” and code 601: “Tail bite/ tail infection”. No further guidance on interpretation of these definitions was given. During meat inspection, tail lesions were seldom registered. In total, 35 pigs (1%) were registered with tail lesions and thereof one pig (0.04%) with code 601. Within batches, 0–9.5% of pigs (median: 1%) were registered with tail lesions at the abattoir.

In the statistical analysis, we compared the prevalence of severe lesions registered in the herds with the prevalence of tail lesions (code 600 + code 601) registered at the abattoir. Comparison was done by linear regression of proportions registered in the herds versus proportions in the abattoir. Results are presented as a regression line with 95% confidence bands. In order to explore the presence of a systematic relationship, we applied a leave-one-batch-out internal cross-validation of prediction of the proportion of severe clinical lesions from the proportion of lesions reported at the abattoir. From this, we report the mean absolute difference between predicted and observed proportions. In addition, for comparison with previous studies; the coefficient of determination (r^2^) was calculated. Data management, plotting and analyses were carried out using R version 3.4.4 [7].

Figure 2 shows a plot of the prevalence of severe lesions observed in the herds within the 24 batches against the prevalence of abattoir tail lesions. It appears from the figure, that the detection rate at the abattoir generally was markedly lower than the detection rate in the herds (note the difference in axis range). Furthermore, as demonstrated by the figure, no systematic relationship seemed to exist. The model was not able to predict prevalence of severe clinical tail lesions with a reasonable precision (r^2^ = 0.006). The mean absolute difference between the predicted and the observed proportion was 9%-points.

**Fig. 2:**
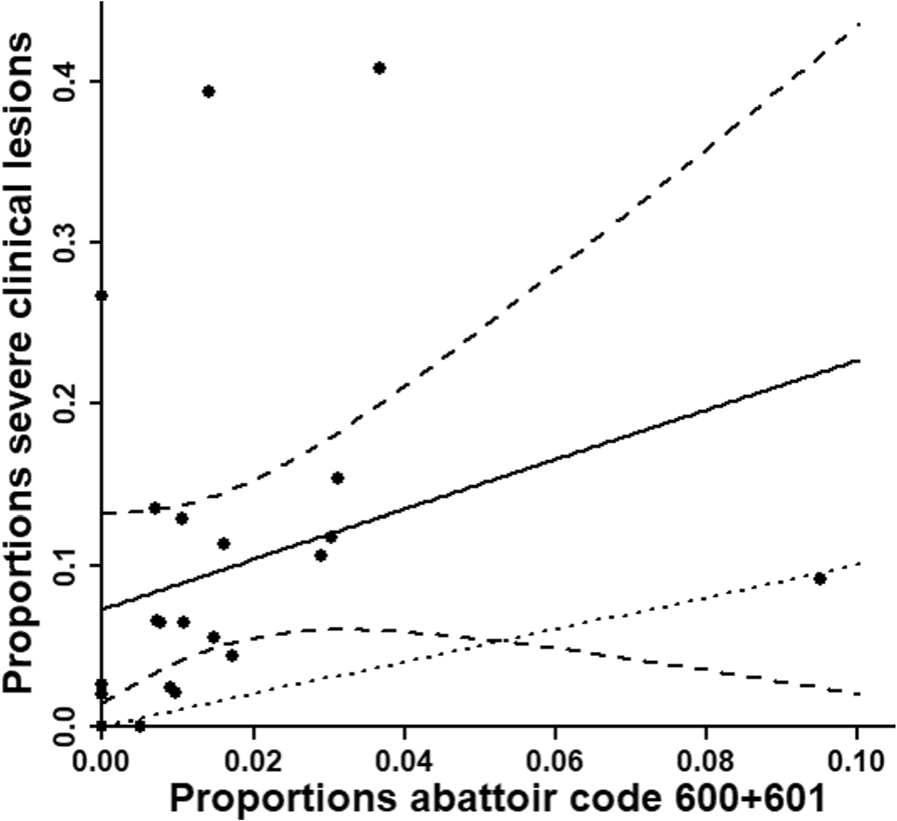
Agreement between Severe lesions detected by clinical examination and abattoir registrations. Severe tail lesions registered in the herds (Y-axis) vs tail lesions registered at the abattoir (code 600 and 601) (X-axis) in 24 batches of slaughter pigs (bullets). The estimated regression line (solid) with 95% confidence bands (dashed lines) is shown. The identity line (dotted line with slope 1) indicating a perfect agreement is also shown

Altogether, our results indicate that the prevalence of tail lesions registered by routine meat inspection is generally lower than the prevalence recorded by clinical examination. Furthermore, there is a non-linear and unsystematic relationship between abattoir and herd- registrations. A challenge in using abattoir registrations as an indicator of the tail bite prevalence in herds is the fact that lesions heal with time. Thus, tail bite problems occurring early in the rearing period are not as apparent at slaughter as problems occurring late. The vast majority of lesions in this study were chronic in nature, and this probably explains the apparent underreporting at the abattoir.

Previous studies confirm that performing clinical registrations on tails of live animals in stables and on carcasses at the abattoir does not give the same result. Even studies where researchers performed detailed abattoir registrations found weak to moderate correlations [4, 6] between tail lesion prevalence in the stable and tail lesions registered at the abattoir. In these studies, tail lesions were scored on a detailed score not applicable for inspectors working at a moving slaughter line, which was the context in the current study. Interestingly, and a bit in contrast to the results of our study, Carroll et al. [8] found that scalding and dehairing of tails increased the visibility of lesions, when using the same scale for scoring before and after scalding. Problems on under-reporting of lesions may be resolved by clear instructions on the appearance of normal undocked tails and allowing more time for meat inspection. Vom Brocke et al. [9] compared results from meat inspection with an assessment from photos taken at the abattoir. The observed prevalence of tail necrosis (definition not given in the manuscript) was 0.22% when based on meat inspection vs. 0.69% when based on pictures, perhaps indicating, that given more time, a better performance can be obtained. All the previous studies referred to here, were performed on docked pigs.

The results of the present study demonstrate that currently recorded abattoir data does not seem a reliable indicator of tail bite problems in herds with undocked pigs and therefore not useful as a general monitoring tool for farmers. Improved and cost effective methods for abattoir detection of tail lesions are needed if abattoir registrations are to be used as a valid monitoring tool. A 3D vision-based recording system is currently under development and may have a potential for reliable and cost-effective monitoring of tail lesions [10].

## Data Availability

The datasets generated and/or analysed during the current study are not publicly available because of the risk of compromising private policy but are available from the corresponding author on request.

## Footnotes

1 Herds with outdoor farrowing, later weaning (5–7 weeks of age), permanent outdoor access, decreased stocking density compared to conventional herds and provision of bedding material and roughage.
